# Locally advanced breast cancer treated with neoadjuvant chemotherapy and adjuvant radiotherapy: a retrospective cohort analysis

**DOI:** 10.1186/s12885-019-5499-2

**Published:** 2019-04-03

**Authors:** Jonathan Klein, William Tran, Elyse Watkins, Danny Vesprini, Frances C. Wright, Nicole J. Look Hong, Sonal Ghandi, Alex Kiss, Gregory J. Czarnota

**Affiliations:** 1 Department of Radiation Oncology, Faculty of Medicine, University of Toronto, Toronto, Canada; 2 Department of Radiation Oncology, Sunnybrook Health Sciences Centre, 2075 Bayview Avenue, T2-067, Toronto, Ontario M4N 3M5 Canada; 3 Department of Medical Biophysics, University of Toronto, Toronto, Canada; 4 Department of Surgery, Sunnybrook Health Sciences Centre, and Faculty of Medicine, University of Toronto, Toronto, Canada; 5 Division of Internal Medicine, Department of Medicine, Sunnybrook Health Sciences Centre, Toronto, Canada; 6 Department of Medicine, Faculty of Medicine, University of Toronto, Toronto, Canada; 7 Institute of Clinical Evaluative Sciences, Sunnybrook Health Sciences Centre, Toronto, Canada

**Keywords:** Breast cancer, Neoadjuvant therapy, Pathologic response, Radiotherapy, Response, Chevallier

## Abstract

**Background:**

Neoadjuvant chemotherapy (NAC) is increasingly used to treat locally advanced breast cancer (LABC). Improved response to NAC correlates with better survival outcomes. The dual purpose of this study is to report recurrence and survival outcomes for LABC patients treated with NAC, surgery and adjuvant radiotherapy and to correlate these outcomes with tumour response after NAC using multiple response assessment methods.

**Methods:**

All LABC patients treated for curative intent with NAC, surgery, and adjuvant radiotherapy at our institute between January 2009 and December 2014 were included for analysis. NAC was mostly anthracycline and taxane-based; radiotherapy consisted of 50 Gy to the breast/chest wall and regional lymph nodes. Response to NAC was categorized using synoptic pathology reports, modified-RECIST and Chevallier scores. Survival curves were generated by the Kaplan-Meier method and compared using the log-rank test.

**Results:**

The cohort included 103 patients nearly equally divided between Stage II (*n* = 53) and Stage III (*n* = 50). Rates of locoregional control (LRC), recurrence-free survival (RFS), and overall survival (OS) were 99, 98, and 100% at 1 year and 89, 69 and 77% at 5 years, respectively. Responses to NAC did not correlate with LRC (*p > 0.05*) but did correlate with RFS and OS (*p < 0.05*), except that the Chevallier score did not predict RFS (*p = 0.06*). Using bivariate Cox modeling tumour size before (*p = 0.003*) and after (*p < 0.001*) NAC, stage group (*p = 0.05*), and response assessed by synoptic pathology (*p = 0.05*), modified-RECIST (*p = 0.001*), and Chevallier score (*p = 0.015*) all predicted for RFS. No factors predicted for LRC.

**Conclusion:**

Pathologic response by all tested methods correlated with improved survival but were not associated with decreased LRC.

## Background

Neoadjuvant chemotherapy (NAC) is increasingly used to treat patients with locally advanced breast cancer (LABC) [1–3]. Such regimens can increase rates of breast-conserving therapy (BCT) compared with post-operative chemotherapy [2] and may minimize the need for aggressive nodal surgery with axillary lymph node dissection [4, 5]. Other purported advantages include in vivo tumour response assessment and prognostication based on degree of response. Patients with HER2-receptor positive or triple-negative disease may also benefit from early treatment of distant micrometastases due to increased metastatic potential of these disease types [6, 7]. Despite these potential advantages, NAC has not demonstrated improved survival over adjuvant chemotherapy in randomized trials [8–11].

The NSABP B-18 and B-27 trials delivered anthracycline-based NAC (and included taxanes in B-27). Patients who underwent breast conserving surgery (BCS) after NAC received whole breast radiation therapy (RT) alone, while mastectomy patients did not receive RT. These trials demonstrated locoregional recurrence rates of 12.3% for mastectomy patients and 10.3% for BCS patients [12]; overall survival (OS) rates were 75% at 8 years [6]. Subsequent retrospective studies [13, 14] have suggested that adjuvant RT decreases the rate of locoregional recurrence (LRR) and improves survival after NAC and surgery, but this has not been evaluated with randomized trials. Given the absence of level I evidence, routine practice at our centre is to offer radiation therapy after NAC and surgery.

Patients who achieve a pathologic complete response (pCR) to NAC have improved survival compared to patients who do not achieve pCR [8, 15, 16]. Several quantitative and categorical methods have been developed to characterize pathologic response to NAC, including residual cancer burden index (RCBI) [17], the Chevallier score [18], and the Miller-Payne score [19]. Radiologic assessment of tumor size changes based on the Response Evaluation Criteria in Solid Tumours (RECIST) guidelines [20] correlates with pathologic response [21], although MRI may not reliably detect tumours pathologically measured as smaller than 2 cm [22]. For these response criteria, increased local response to the primary tumour from NAC correlates well with improved survival [17, 18, 21, 23].

In this study, response to NAC was measured using three methods. The first used data from synoptic pathology reports prepared by our institutional pathologists. The second, Chevallier score [18], provides a response measurement based on a combination of microscopic and macroscopic assessment of changes in tumour appearance. The third method categorized response radiographically, using RECIST criteria combined with pathologic verification of complete response if suspected radiographically.

This study had two main goals. First, we investigated recurrence and survival outcomes for a cohort of LABC patients treated with NAC, surgery, and RT. Patients treated with all three modalities were selected as this represented the majority of patients treated at our centre and we wanted to ensure that results were reported from as homogeneous a cohort as possible. Second, we assessed tumour response after NAC using synoptic pathology reports, Chevallier score and a modified RECIST criteria-based (MR) response score and correlated these responses with recurrence and survival outcomes in our cohort.

## Methods

### Patients and treatment

Institutional review board approval was obtained prior to data collection and analyses for this retrospective study. Data was collected via electronic medical records for all female breast cancer patients treated with NAC, surgery and radiotherapy with curative intent at our center between January 2009 and December 2014 for whom complete clinical data was available. Clinical tumour (T) staging used physical exam or pre-chemotherapy imaging (MRI, mammogram and/or ultrasound). Pre-chemotherapy clinical nodal (N) assessment consisted of physical exam and imaging. Pre-chemotherapy lymph node involvement was assessed via core or fine needle aspiration biopsy, if available, but was not included in the clinical stage grouping. Stage grouping used the American Joint Committee on Cancer (AJCC) 7th edition staging guidelines.

Prior to beginning NAC, all patients had biopsy-confirmed breast cancer that was either larger than 5 cm, invading into the skin/chest wall, or involving the axillary lymph nodes. Patients received NAC followed by mastectomy or BCT including surgical excision of the axillary lymph nodes. Adjuvant RT delivered 45–50 Gy in 25 fractions to the chest wall (or whole breast, if applicable), axillary and supraclavicular lymph node regions. The RT was delivered via monoisocentric three or four field technique consisting of opposed tangent fields to the breast/chestwall and lymph node coverage with either opposed anterior-posterior fields or anterior field alone. Radiotherapy to the internal mammary lymph node chain and boost RT to the tumour cavity were left to the discretion of the treating radiation oncologist; generally boost RT was offered to patients treated with BCT who were younger than age 50 or had high risk features such as high grade disease or close/positive margins [24]. All patients began RT between three and six weeks after surgery, per our institutional guidelines. Following radiotherapy, patients with estrogen receptor (ER) positive disease were offered hormonal therapy and patients with HER2 receptor overexpression were offered trastuzumab therapy.

### Clinical and pathologic features

Tumours were grouped into molecular subtypes based on hormone and HER2 receptor status, according to the St. Gallen consensus [25]. Subgroups were assigned as follows: Luminal A tumours were positive for ER and progesterone receptor (PR) overexpression, but did not overexpress HER2-neu receptors. Luminal B (HER2-negative) tumours were ER-positive/PR-negative/HER2-negative. Luminal B (HER2-positive) tumours were ER-positive/PR-positive/HER2-positive. HER2+ tumours were ER-negative/PR-negative/HER2-positive. Basal-type tumours were negative for all three receptors. One patient had ER+/PR+/HER2- cancer with Ki67 level > 80%; this patient was assigned to the Luminal B (HER2-negative) group. For the purposes of PR status, “positive” was defined as > 20% overexpression [25–27].

Response to NAC for all patients was categorized using three methods. The first (hereafter referred to as “standard pathology”) reviewed synoptic pathology reports available for each patient produced using the American Joint Committee on Cancer (AJCC) 8th edition criteria for staging and synoptic reporting [28]. At the end of each report, the assessing pathologist categorized the observed response as i) “complete responder,” defined as absence of invasive breast cancer in the breast tissue or axillary lymph nodes, ii) “partial responder”, denoted by probable or definite response in the synoptic report, or iii) “non-responder,” who had no probable or definite response [29].

The second method was the Chevallier score, based on combined macroscopic and microscopic assessment: Grade 1 denotes complete disappearance of macroscopic or microscopic tumour, grade 2 has carcinoma in situ but no remaining invasive tumour or lymph node involvement, grade 3 indicates invasive disease present with stromal alteration and grade 4 indicates little or no modification of tumoural appearance [18].

The third method was the MR system, which categorizes response based on tumor size using RECIST criteria. Size was assessed pre-chemotherapy by imaging or physical exam; the proportion of measurements by different modalities is shown in Table 1. Post-chemotherapy size was assessed using the surgical pathology specimen. A score of 1 indicates no reduction in overall size, score of 2 indicates up to 30% decrease in size, score of 3 indicates a decrease in size of 30–90%, score of 4 indicates greater than 90% but less than complete response and score 5 indicates a complete absence of invasive tumour radiographically and confirmation of no remaining tumour by standard pathology. This system was modified from the RECIST criteria in order to permit a better discretization of response while recognizing patients that were almost complete responders but whose residual disease may not be reliably detected by MRI or other imaging tests.

**Table 1:** Pre-treatment patient characteristics

Category		Number	Percentage
Age (mean ± SD)		49.4 ± 10.7 years	
Pretreatment size (mean ± SD)		5.6 ± 2.8 cm	
Chemotherapy regimen			
	Dose dense AC-T	50	48.6
	AC-T	18	17.5
	FEC-D	29	28.2
	ED	1	1.0
	TC	4	3.9
Estrogen Receptor Status			
	Positive	66	64.1
	Negative	37	35.9
Progesterone receptor status			
	Positive	58	56.3
	Negative	45	43.7
HER2 receptor status			
	Positive	32	31.1
	Negative	71	68.9
Classification			
	Luminal A	30	29.1
	Luminal B (HER2-negative)	17	16.5
	Luminal B (HER2-positive)	18	17.5
	HER2 positive	13	12.6
	Triple-negative	24	23.3
Laterality			
	Left	47	45.6
	Right	55	35.4
	Bilateral	1	1.0
Menopausal status			
	Pre-menopausal	57	55.3
	Post-menopausal	37	35.9
	Peri-menopausal	7	6.8
	Not available	2	1.9
Histology			
	IDC	97	94.2
	ILC	3	2.9
	IMC	1	1.0
	Mixed ILC/IDC	2	1.9
Lymphovascular invasion			
	Negative	50	48.5
	Positive	45	43.7
	Not available	8	7.8
T (tumor) stage			
	T1	2	1.9
	T2	44	42.7
	T3	46	44.7
	T4	11	10.7
Clinical N (nodal) stage			
	N0	28	27.2
	N1	62	60.2
	N2	12	11.7
	N3	1	1.0
Stage group			
	IIA	16	15.5
	IIB	37	35.9
	IIIA	38	6.9
	IIIB	11	10.7
	IIIC	1	1.0
Pathologic N (nodal) status			
	Negative	10	9.7
	Positive	80	77.7
	N/A	13	12.6

Abbreviations: *A* doxorucibin (Adriamycin), *C* cyclophosphamide, *D* docetaxel, *E* epirubicin, *F* 5-fluorouracil, *IDC* invasive ductal carcinoma, *ILC* invasive lobular carcinoma, *IMC* invasive mammary carcinoma, *SD* standard deviation, *T* paclitaxel (Taxol)

### Survival endpoints

Three survival parameters were calculated: locoregional control (LRC) was defined as survival without LRR defined as recurrence in the breast, chest wall, or axillary, supraclavicular, or internal mammary lymph nodes; recurrence-free survival (RFS) was defined as survival without LRR or distant metastasis; and OS was defined as survival without death. Documentation of recurrences was based on available medical records, including dictated clinical notes and radiological studies. Time of recurrence was defined as the date when definitive recurrence or disease progression was first noted in a clinical or radiology report. Death was determined via clinical notes, hospital records and obituary search. The start date of NAC was used as a surrogate for the date of diagnosis; survival times and time to recurrence were calculated from this date.

### Statistical analysis

All patients in the cohort had their response categorized using all three methods. Each method was then assessed to determine if they were predictive of LRC, RFS, and OS. Survival curves were generated by the Kaplan-Meier method and survival differences between groups were compared via the log-rank test. Cox proportional hazard regression modeling was used to analyze factors contributing to survival rates. The association between MP and Chevallier scores (which can be treated as ordinal) was examined using Spearman correlation. To assess the correlation between standard pathology response (categorical variable) and MP and Chevallier scores, a regression analysis was carried out. Analysis was performed using SAS Version 9.4 (SAS Institute, Cary, NC, USA).

## Results

Our cohort consisted of 103 patients and the median follow up was 45.6 months. Median age was 46.7 years old. An additional three patients were identified who otherwise met our inclusion criteria but were not included in the analysis due to lack of clinical follow up after completion of therapy. Pre-treatment patient and disease characteristics are shown in Table 1. Pathologic characteristics after NAC are shown in Table 2. All patients received NAC including a taxane and anthracyline, except for four patients who received paclitaxel and cyclophosphamide.

**Table 2:** Pathologic characteristics

Category		Number	Percentage
Standard pathology			
	No Response	14	13.6
	Partial Response	63	61.2
	Complete response	26	25.2
Miller-Payne score			
	1	6	5.8
	2	9	8.7
	3	43	41.7
	4	14	13.6
	5	31	30.1
Chevallier score			
	1	22	21.4
	2	11	10.7
	3	61	59.2
	4	9	8.7
Nottingham grade			
	1	6	5.8
	2	45	43.7
	3	10	9.7
	Not available	42	40.8

There were 23 patients (21.3%) who experienced disease recurrence. One patient had synchronous regional (lymph node) and distant recurrence. Seven patients (6.8%) had LRR only; five of these (4.8%) were in the ipsilateral chest wall or intact breast, the other two (1.9%) had recurrences in the axillary lymph nodes. Of 15 patients (14.6%) whose first episode of recurrence was distant (without LRR), six had recurrence in the lungs, nine in the bones, six in the liver, and four in the brain. Median time to recurrence was 22.5 months. During the analysis period, 14 deaths (13.6% of patients) were recorded.

**Fig. 1:**
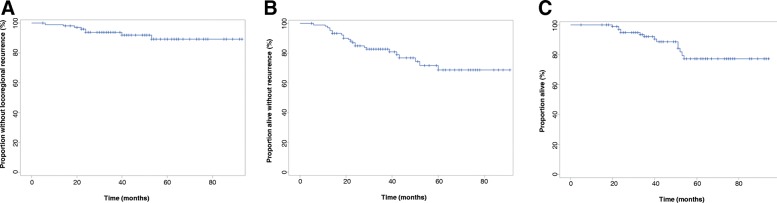
Outcomes for entire patient cohort. **a** Locoregional control **b**) Recurrence-free survival C) Overall survival

For the entire cohort, LRC rates were 99% at 1 year, 94% at 2 years, and 89% at 5-years. Rates of RFS rates were 98% at 1 year, 85% at 2 years, and 69% at 5-years. Rates of OS were 100% at 1 year, 95% at 2 years, and 77% at 5-years. Kaplan-Meier curves with these results are shown in Fig. 1.

**Fig. 2:**
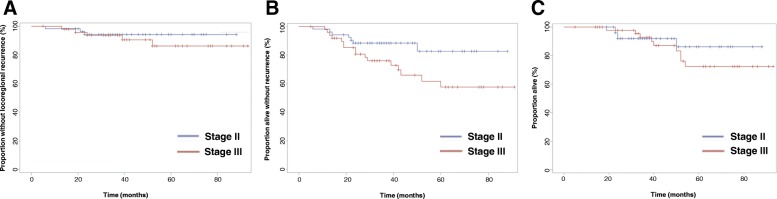
Outcomes for patients divided by Stage II and Stage III disease. **a** Locoregional control **b**) Recurrence-free survival C) Overall survival

The cohort was nearly equally divided between patients with stage II (51%) and stage III (49%) disease. No significant difference in LRR was seen between these two groups (*p = 0.51*). A significant RFS difference (*p = 0.04*) was seen, with 5-year RFS for stage II patients of 83 and 58% for stage III patients. 5-year OS rates were 86% for stage II patients and 72% for stage III patients, although this difference was not statistically significant (*p = 0.46*). Survival curves by stage are shown in Fig. 2. Different molecular subtypes did not demonstrate differences in LRR (*p = 0.11*), RFS (*p = 0.45*), or OS (*p = 0.60*).

### Pathologic response

**Fig. 3:**
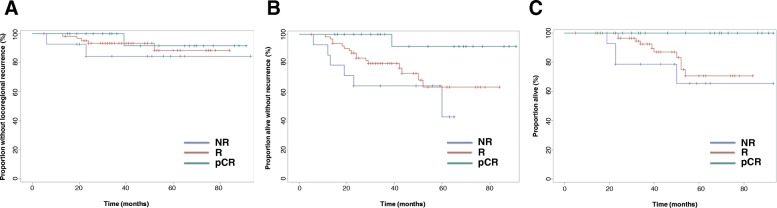
Outcomes for patient cohort subdivided by response to neoadjuvant chemotherapy assessed by standard pathology. **a** Locoregional control **b**) Recurrence-free survival **c**) Overall survival. NR = non-responder, R = responder, pCR = pathologic complete response

Kaplan-Meier curves for recurrence and survival outcomes with patients divided by standard pathology response category are shown in Fig. 3. No significant differences were seen in LRC rates (*p = 0.48*), but significant differences were observed for RFS (*p = 0.015*) and OS (*p = 0.015*). No patients achieving a pCR died within 5-year of treatment completion.

**Fig. 4:**
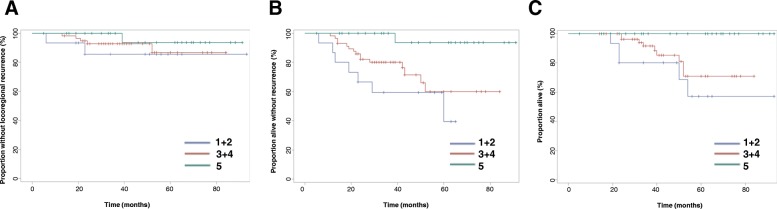
Outcomes for patient cohort subdivided by modified-RECIST criteria (MR) grouping. **a** Locoregional control **b**) Recurrence-free survival **c**) Overall survival. MR score 1 and 2 (MR 1 + 2) are grouped together as “non-responders,” MR 3 and 4 (MR 3 + 4) are “partial responders” and MR 5 is “complete response”

For patients divided by MR score, local recurrence rates were not significantly different (*p = 0.45*), but significant differences were observed for RFS (*p = 0.003*) and OS (*p = 0.014*), as shown in Fig. 4. There were no deaths reported among patients classified as MR 5. Note that due to small numbers of patients with certain MR scores, patients were grouped as follows: MR 1 and 2 were grouped together as “non-responders,” 3 and 4 as “responders” and MR 5 patients were “complete responders.”

**Fig. 5:**
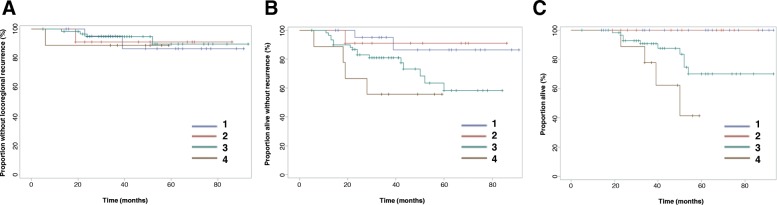
Outcomes for patient cohort subdivided by Chevallier score (CS). **a** Locoregional control **b**) Recurrence-free survival **c**) Overall survival. Only 1 patient in the cohort had a Chevallier score of 2; thus, this subject was censored

No difference in LRR rates (*p = 0.96*) was seen between patients grouped by Chevallier score, as shown in Fig. 5. Differences in RFS reached borderline significance (*p = 0.06*). Nevertheless, significant differences in OS were observed (*p = 0.005*), with no deaths reported at 5 years for patients with Chevallier score of 1 or 2.

Pathologic complete response rates, as determined by each assessment method, are shown in Table 3. Outcomes at 1 and 5 years with patients divided by response to NAC are summarized in Table 4.

**Table 3:** Complete Response Rates

		Luminal A	Luminal B (HER2-negative)	Luminal B (Her2-positive)	HER2-positive	Triple negative
Number of patients with molecular subtype		30	17	18	13	24
Percent of patients with subtype achieving:						
	Standard pathology pCR (%)	3.3	11.7	33.3	46.2	41.7
	Chevallier 1 (%)	3.3	23.5	27.8	38.5	33.3
	MR 5 (%)	3.3	17.6	56.3	61.5	45.8

Abbreviations: *pCR* pathologic complete response, *MR* Modified-RECIST

**Table 4:** Outcomes by response measurement

		LRC			RFS			OS		
		1 year (%)	5 years (%)	*p*-value	1 year (%)	5 years (%)	*p*-value	1 year (%)	5 years (%)	*p*-value
Standard pathology				0.48			***0.015***			***0.015***
	pCR	100	92		100	92		100	100	
	PR	100	88		98	63		100	71	
	NR	94	84		86	43		100	65	
Chevallier										
	1 (pCR)	100	86	0.96	100	86	***0.06***	100	100	***0.005***
	2	100	91		100	91		100	100	
	3	100	90		97	58		100	70	
	4	100	N/A		89	N/A		100	N/A	
Modified-RECIST										
	5 (pCR)	100	94	0.45	100	94	***0.003***	100	100	***0.011***
	3 + 4	100	87		98	60		100	71	
	1 + 2	93	86		87	40		100	57	

Abbreviations: *LRC* locoregional control rate, *NR* no response, *OS* overall survival, *pCR* pathologic complete response, *PR* partial response, *RECIST* Response Evaluation Criteria in Solid Tumors, *RFS* recurrence-free survival

Standard pathology response was significantly associated with both Chevallier (*p* < 0.001) and MR score (p < 0.001). The Spearman correlation between MR and Chevallier showed a strong negative association (r = − 0.81, *p* < 0.0001). Regression analysis showed bivariate correlation between MR and standard pathology was 0.874 and between Chevallier and standard pathology was 0.775.

### Predictors of recurrence and survival

Using a bivariate Cox model, we did not identify any significant predictors of LRR. We were not able to perform multivariable analysis due to the small number of events in our sample. Tumour size (both before and after NAC), pre-treatment stage group, and pathological response assessed by all three methods (standard pathology, a MR score, and Chevallier) significantly predicted for RFS. Analysis of OS is not reported due to the small number of deaths in our cohort. The results of this analysis are shown in Table 5.

**Table 5:** Bivariate analysis with Cox proportional hazard model

		Local recurrence			Recurrence-free survival		
Variable		Hazard ratio	95% CI	*p*-value	Hazard ratio	95% CI	*p*-value
Nottigham grade	2 vs. 3	0.30	0.05–1.81	0.43	0.67	0.24–1.88	0.75
Menopausal status	Post vs. pre	0.25	0.03–2.10	0.44	1.32	0.57–3.06	0.81
LVI	No vs. Yes	0.69	0.15–3.08	0.63	0.48	0.19–1.22	0.12
Pathological nodal status	No vs. yes	1.33	0.16–11.03	0.79	0.41	0.05–3.05	0.38
Tumour size before surgery		1.09	0.86–1.38	0.47	1.24	1.08–1.42	**0.003**
Tumour size at surgery		1.03	0.85–1.24	0.77	1.18	1.09–1.29	**< 0.001**
Miller-Payne score		0.74	0.42–1.32	0.31	0.56	0.4–0.8	**0.001**
Chevallier score		0.96	0.46–2.02	0.92	2.01	1.14–3.53	**0.015**
Standard pathology response				0.51			**0.05**
	NR vs. pCR	3.98	0.36–44.0		13.29	1.6–110.5	
	PR vs. pCR	2.07	0.24–17.7		7.43	0.99–56.1	
Pretreatment stage group				0.51			**0.05**
	II vs. III	0.62	0.15–2.61		0.41	0.17–1.0	

Abbreviations: *CI* confidence interval, *LVI* lymphovascular invasion, *NR* no response, *pCR* pathological complete response, *PR* partial response

## Discussion

All 103 patients in our cohort were evaluated for primary tumour response to NAC using three methods: standard pathology, MR score, and Chevallier score. Our results suggest that breast cancer treated with NAC followed by surgery and RT is associated with low rates of LRR (approximately 10% at 5 years) and relatively high OS (greater than 75% at 5 years). Rates of RFS and OS were significantly improved with increasing response to NAC, but LRR was not affected. Distant recurrence alone (15 patients) was twice as common as LRR alone (7 patients), similar to previously reported results of RT after NAC and surgery [30]. Molecular subtype did not significantly affect recurrence or survival rates.

Rates of 5-year RFS for pCR, MR score 5, and Chevallier score 1 were 92%, 93%, and 86% respectively, while 5-year OS was 100% for all three groups. The similarity in outcomes suggests that all are valid methods for predicting outcomes after NAC, especially for those patients who achieve pCR. Patients grouped by MR score of 3 or 4 (denoting “partial responder”) or 1 or 2 (“non-responder”) tended to have worse outcomes than similar categories using standard pathology.

### Tumour response assessment after neoadjuvant chemotherapy

In our cohort, the three response methods employed detected similar rates of pCR: 25.2% of patients achieved pCR by standard pathology, 30.1% achieved MR score of 5 and 21.4% had Chevallier score of 1. These rates are similar to the 26.1% pCR rate in NSABP B-27. Over 95% of patients received anthracycline and taxane-based chemotherapy, similar to that given to NSABP B-27 patients [9].

All three treatment response methods showed similar predictive abilities for clinical outcomes. Using bivariate Cox modeling, all three methods were predictive of RFS, but none were predictive of LRR. Using Kaplan-Meier methodology, when patients were stratified by standard response assessment and MP, response to NAC did not predict for LRR but did predict both RFS and OS. Chevallier score did not predict LRR and did predict for OS; a borderline (*p = 0.06*) significant predictive ability was measured for RFS. Strong correlations between categorizations using the different methods were also demonstrated.

These results are reassuring, suggesting that the three methods (two pathologically derived and one based on radiographic findings) all serve as adequate predictors of response and ultimate clinical outcomes. The results agree with those of Romero et al. who reported that RCBI and RECIST-based assessments both predicted for RFS in a cohort of 151 patients [21]. This cohort was similar to ours in proportion of tumours that were hormone-receptor positive (58% versus 64% in our cohort), although a higher proportion had Stage III disease (69% versus 49% in our cohort).

Recurrence and survival results in our cohort were similar to those reported in the initial publication by Ogston et al. that validated the Miller-Payne pathology scoring system [19]. The initial cohort divided responses into three groups based on survival outcomes: Patients with Miller-Payne score 1 or 2 in the original cohort had 5-year RFS of 55–60%. For patients with Miller-Payne score 3 or 4, 5-year RFS in the initial cohort was 65–75%. For patients with Miller-Payne score 5 (indicating complete response), 5-year RFS was 95% in the initial cohort and 94% in our cohort. We were unable to conduct Miller-Payne scoring here but used the MR size-based method in order to assess potentially the same magnitudes of response in general terms. Our cohort had a lower proportion of node-negative patients (27%) compared with the initial cohort of Ogston et al. (57%). Lymph node involvement is a strong predictor of LRR and survival with breast cancer [31, 32], which may explain why patients with similar magnitudes of response had lower survival rates in our cohort than in the initial cohort. Our results agree with existing literature, and suggest that multiple established pathologic and radiographic response measurements can provide reliable prognostic value for patients and clinicians.

Different pathologic subtypes of breast cancer may behave differently in response to NAC. Von Minckwitz et al. studied responses to NAC by different pathologic subtypes, in a cohort of over 6000 patients. They found that HER2-positive and triple-negative cancers achieved the highest pCR rates with Luminal A patients experiencing the lowest. If pCR was defined to allow residual in situ disease, pCR rates were 8.9, 51, and 35.8% for Luminal A, HER2-positive, and triple-negative, respectively [29]. Our results closely match these findings, with pCR rates of 6.5, 42.9, and 41.7%, respectively, if in situ disease was allowed within the pCR definition.

Lee et al. studied over 500 patients and assessed response by both “relative” methods (i.e. those that compare size or cellularity of post-NAC samples with pre-NAC data) such as MR score and “absolute” methods (i.e. those that use only the post-NAC sample) such as RCBI. They concluded that for triple negative cancer, all response assessment methods can predict for disease-free survival. However, for hormone-receptor positive, HER2-receptor negative disease, only absolute methods had prognostic value [33]. Our cohort was not large enough to study the prognostic value of response only among patients of a single pathologic subgroup.

All three response assessment methods we studied showed the highest rates of RFS and OS among patients who achieved pCR. The NSABP B-18 and B-27 studies compared outcomes after randomizing patients with operable breast cancer between NAC and post-operative chemotherapy. Patients in the NSABP B-18 trial received AC chemotherapy (doxorubicin and cyclophosphamide), while the B-27 study used AC plus docetaxel. Hazard ratios for recurrence and death for patients not achieving pCR compared with those who did not were 0.47 and 0.32, respectively, after 15-year follow-up in the B-18 trial and 0.49 and 0.36, respectively, after 8 year follow-up in B-27. Neither study demonstrated statistically significant differences between the NAC and adjuvant chemotherapy groups for either disease-free survival or OS [8–10].

Our results support the hypothesis that pCR (as determined by all methods studied) is predictive of RFS and OS, even when RT is given after surgery (which was not given in the B-18 and B-27 studies). However, based on all three response methods assessed in this study, pCR does not appear to be predictive of LRR when RT is given. In the combined B-18 and B-27 cohort, predictors of LRR on multivariable analysis were: age at randomization, tumor size before NAC, clinical nodal status before NAC, and pCR after NAC. In our cohort, these same factors predicted for RFS (with pCR assessment by all three methods). However, we did not identify any factors that significantly predicted for LRR in our cohort. Our data support the NSABP findings that these factors are significant prognostic markers, but the RT that our patients received seems to have reduced these factors’ influence on LRR rates [12].

### Clinical outcomes after neoadjuvant chemotherapy, surgery, and radiotherapy

The role of adjuvant RT after NAC and surgery has not been evaluated in randomized trials but is generally recommended for patients with cT3-T4 and/or N2-N3 disease [34, 35]. It may also be considered for patients with smaller, but still lymph node-positive, disease, especially those with high-risk factors such as young age or triple-negative biology [36]. Retrospective analyses suggest that adjuvant RT benefits patients treated with NAC for advanced breast cancer. Huang et al. [37] compared 542 patients who received NAC, surgery, and adjuvant RT with 134 patients who did not receive RT. The RT cohort had more advanced disease (73% pre-treatment stage III and 10% stage IV) than those who did not (46% stage III and 4% stage IV). Rates of LRR were nonetheless significantly lower for RT patients (11% versus 22%; *p = 0.0001*), and RT significantly improved cause-specific survival for patients with stage IIIB or IV disease, clinical T4 tumours, and four or more positive lymph nodes. McGuire et al. [10] studied 106 patients without inflammatory breast cancer, all of whom achieved pCR after NAC (92% included an anthracycline and 38% included a taxane in their NAC). Among the 67% of patients who had stage III disease at diagnosis, the 10-year LRR rate was 33.3% without RT and 7.3% with RT (*p = 0.04*) and RT also improved OS (*p = 0.0017*). No recurrence benefit was seen for patients with stage I and II breast cancer who achieved pCR however.

Our cohort had a similar stage distribution (51.5% stage II, 48.5% stage III) to these other retrospective reports. Our cohort's LRR rate (approximately 10% recurrence at 5 years) agrees closely with the results reported by Huang and McGuire for patients who received adjuvant RT. In the McGuire study, 5-year OS among patients who received RT was over 80%. Patients in our cohort who achieved a pCR had 5-year OS of 100% compared with 71% for partial responders and 65% for non-responders. The low rates of LRR seen in our cohort (particularly among patients who achieved pCR) mirrors the low rate of LRR in previous retrospective analyses. These retrospective cohorts together appear to support the routine use of adjuvant RT after NAC for clinical stage III disease, even after pCR. The question of RT after pCR is the subject of ongoing trials, including NSABP B-51 which is randomizing patients with T1–3, N1 disease between nodal RT and no nodal RT.

Buccholz et al. [38] reported on 150 patients treated with NAC and mastectomy only. The plurality of patients (48%) had stage III disease (43% stage II, 7% stage IV). The reported 5-year LRR rate in this study was 27%, higher than the 8% in our cohort. Although our cohort did not include any stage IV patients, the relative proportion of patients with stage II and stage III disease was similar between our cohort and that of Buccholz et al. However, our patients all received post-operative RT, which may account for the lower LRR observed in our cohort. Buccholz et al. reported that pretreatment T stage and clinical stage group, lymph node involvement, and tamoxifen use predicted for LRR, while we did not identify identify any predictor for LRR on bivariate analysis, including nodal involvement or pretreatment stage. This difference may also reflect the effect of radiotherapy at reducing LRR risk.

In the NSABP B-27 trial, LRR at 10 years for patients who received AC-T chemotherapy was 9.5%. In the combined NSABP B-18 and B-27 dataset, the 10 year LRR rate was 11.1% (8.4% local and 2.7% regional recurrence). Local recurrences accounted for 71% of 10-year LRR in patients treated with mastectomy and for 79% of 10-year LRR in patients treated with lumpectomy plus breast XRT [12]. Rates of LRR in our cohort were similar at 5 years to those reported at 10 years in NSABP B-27, although the patients in our cohort received postoperative radiotherapy and the NSABP patients did not. Patients in our cohort also had approximately twice the risk of distant recurrence as LRR, which is the opposite of the NSABP trials. These differences may arise because 70% of patients in our cohort having involved lymph nodes compared with around 30% in NSABP B-27, and patients with N2 staged disease being excluded from B-27 (13% of our patients had N2–3 disease). Thus, patients in our cohort likely had more aggressive cancer, on average, and a higher recurrence risk than the NSABP cohort, which may explain the elevated LRR in our cohort despite receiving adjuvant RT [12].

### Strengths and limitations

To our knowledge, this study represents the largest North American cohort to date with response to NAC assessed using multiple pathologic scoring systems, which represents a major strength. We observed strong correlations between all three response assessment methods and similar clinical outcomes between pathological pCR, MR-5 and Chevallier-1 (all indicating complete response). These results suggest that all three methods are valid predictors of clinical outcomes when assessing completeness of response. Our study also presents a cohort of relatively homogeneous patients who all received NAC followed by surgery and RT.

Limitations include the study’s retrospective nature, which depends on the completeness and accuracy of the information in clinical notes. Ki67 levels were generally not available, limiting our ability to distinguish between Luminal A and Luminal B (HER2-negative) cancers; however, this likely did not alter our conclusions.

Three patients who were otherwise eligible were excluded from the analysis due to lack of follow up. It is possible that these patients were lost to follow up due to poor outcomes (e.g. morbidity or death), which could bias our results. However, the number of such patients was small compared to the studied cohort size.

We did not have a sufficient number of deaths to study predictors of OS. Longer follow up may allow for more recurrences and deaths to be observed and late recurrence may be more likely in patients with certain outcomes (e.g. non-responders, aggressive molecular subtypes such as triple-negative). We also could not combine pathologic response assessment with molecular subtypes as was done by Lee et al. [26]. A larger sample would allow for such subgroups to be studied, which could provide further data to improve breast cancer prognostics.

## Conclusion

Local recurrence rates after NAC, surgery, and RT are low and the dominant risk for patients is distant recurrence disease. Pathologic complete response, as measured by different pathologic and radiologic methods, is associated with improved survival for these patients but may not be associated with decreased local recurrence. Our data supports the use of adjuvant RT after NAC, but this question remains the subject of ongoing prospective trials.

## References

[1] Mougalian SS, Soulos PR, Killelea BK, Lannin DR, Abu-Khalaf MM, DiGiovanna MP, Sanft TB, Pusztai L, Gross CP, Chagpar AB (2015). Use of neoadjuvant chemotherapy for patients with stage I to III breast cancer in the United States. Cancer.

[2] Killelea BK, Yang VQ, Mougalian S, Horowitz NR, Pusztai L, Chagpar AB, Lannin DR (2015). Neoadjuvant chemotherapy for breast cancer increases the rate of breast conservation: results from the National Cancer Database. J Am Coll Surg.

[3] Puig CA, Hoskin TL, Day CN, Habermann EB, Boughey JC (2017). National Trends in the use of neoadjuvant chemotherapy for hormone receptor-negative breast Cancer: a National Cancer Data Base Study. Ann Surg Oncol.

[4] M P, M M (2017). Axillary nodal management following neoadjuvant chemotherapy: a review. JAMA Oncol.

[5] Hunt KK, Yi M, Mittendorf EA, Guerrero C, Babiera GV, Bedrosian I (2009). Sentinel lymph node surgery after neoadjuvant chemotherapy is accurate and reduces the need for axillary dissection in breast cancer patients. Ann Surg.

[6] Anders CK, Carey LA (2009). Biology, metastatic patterns, and treatment of patients with triple-negative breast cancer. Clin Breast Cancer.

[7] Wahba HA, El-Hadaad HA (2015). Current approaches in treatment of triple-negative breast cancer. Cancer Biol Med.

[8] Rastogi P, Anderson SJ, Bear HD, Geyer CE, Kahlenberg MS, Robidoux A (2008). Preoperative chemotherapy: updates of National Surgical Adjuvant Breast and bowel project protocols B-18 and B-27. J Clin Oncol.

[9] Bear HD, Anderson S, Smith RE, Geyer CE, Mamounas EP, Fisher B (2006). Sequential preoperative or postoperative docetaxel added to preoperative doxorubicin plus cyclophosphamide for operable breast cancer: National Surgical Adjuvant Breast and bowel project protocol B-27. J Clin Oncol.

[10] van der Hage JA, van de Velde CJ, Julien JP, Tubiana-Hulin M, Vandervelden C, Duchateau L (2001). Preoperative chemotherapy in primary operable breast cancer: results from the European Organization for Research and Treatment of Cancer trial 10902. J Clin Oncol.

[11] Early Breast Cancer Trialists' Collaborative Group (EBCTCG) (2018). Long-term outcomes for neoadjuvant versus adjuvant chemotherapy in early breast cancer: meta-analysis of individual patient data from ten randomised trials. Lancet Oncol.

[12] Mamounas EP, Anderson SJ, Dignam JJ, Bear HD, Julian TB, Geyer CE (2012). Predictors of locoregional recurrence after neoadjuvant chemotherapy: results from combined analysis of National Surgical Adjuvant Breast and bowel project B-18 and B-27. J Clin Oncol.

[13] McGuire SE, Gonzalez-Angulo AM, Huang EH, Tucker SL, Kau SW, Yu TK (2007). Postmastectomy radiation improves the outcome of patients with locally advanced breast cancer who achieve a pathologic complete response to neoadjuvant chemotherapy. Int J Radiat Oncol Biol Phys.

[14] Nagar H, Boothe D, Ginter PS, Sison C, Vahdat L, Shin S (2015). Disease-free survival according to the use of postmastectomy radiation therapy after neoadjuvant chemotherapy. Clin Breast Cancer.

[15] Kuerer HM, Newman LA, Smith TL, Ames FC, Hunt KK, Dhingra K (1999). Clinical course of breast cancer patients with complete pathologic primary tumor and axillary lymph node response to doxorubicin-based neoadjuvant chemotherapy. J Clin Oncol.

[16] Guarneri V, Broglio K, Kau SW, Cristofanilli M, Buzdar AU, Valero V (2006). Prognostic value of pathologic complete response after primary chemotherapy in relation to hormone receptor status and other factors. J Clin Oncol.

[17] Symmans WF, Peintinger F, Hatzis C, Rajan R, Kuerer H, Valero V (2007). Measurement of residual breast cancer burden to predict survival after neoadjuvant chemotherapy. J Clin Oncol.

[18] Chevallier B, Roche H, Olivier JP, Chollet P, Hurteloup P (1993). Inflammatory breast cancer. Pilot study of intensive induction chemotherapy (FEC-HD) results in a high histologic response rate. Am J Clin Oncol.

[19] Ogston KN, Miller ID, Payne S, Hutcheon AW, Sarkar TK, Smith I (2003). A new histological grading system to assess response of breast cancers to primary chemotherapy: prognostic significance and survival. Breast.

[20] Eisenhauer EA, Therasse P, Bogaerts J, Schwartz LH, Sargent D, Ford R (2009). New response evaluation criteria in solid tumours: revised RECIST guideline (version 1.1). Eur J Cancer.

[21] Romero A, García-Sáenz JA, Fuentes-Ferrer M, López Garcia-Asenjo JA, Furió V, Román JM (2013). Correlation between response to neoadjuvant chemotherapy and survival in locally advanced breast cancer patients. Ann Oncol.

[22] Marinovich ML, Macaskill P, Irwig L, Sardanelli F, Mamounas E, von Minckwitz G (2015). Agreement between MRI and pathologic breast tumor size after neoadjuvant chemotherapy, and comparison with alternative tests: individual patient data meta-analysis. BMC Cancer.

[23] Penault-Llorca D, Abrial C, Raoelfils I, Cayre A, Mouret-Reynier MA, Leheurter M (2008). Comparison of the prognostic significance of Chevallier and Sataloff's pathologic classifications after neoadjuvant chemotherapy of operable breast cancer. Hum Pathol.

[24] Poortmans P (2013). Optimal approach in early breast cancer: radiation therapy. EJC Suppl.

[25] Goldhirsch A, Wood WC, Coates AS, Gelber RD, Thurlimann B, Senn HJ (2011). Strategies for subtypes--dealing with the diversity of breast cancer: highlights of the St. Gallen international expert consensus on the primary therapy of early breast Cancer 2011. Ann Oncol.

[26] Senn HJ (2013). St. Gallen consensus 2013: optimizing and personalizing primary curative therapy of breast cancer worldwide. Breast Care (Basel).

[27] Prat A, Cheang MC, Martín M, Parker JS, Carrasco E, Caballero R (2013). Prognostic significance of progesterone receptor-positive tumor cells within immunohistochemically defined luminal a breast cancer. J Clin Oncol.

[28] Edge S, Byrd D, Comptom C (2010). AJCC Cancer staging manual.

[29] von Minckwitz G, Untch M, Blohmer JU, Costa SD, Eidtmann H, Fasching PA (2012). Definition and impact of pathologic complete response on prognosis after neoadjuvant chemotherapy in various intrinsic breast cancer subtypes. J Clin Oncol.

[30] White R, Dinneen T, Makris A (2016). Local radiotherapy alone following neoadjuvant chemotherapy and surgery in combined clinical stage II and III breast cancer. Radiat Oncol.

[31] Cianfrocca M, Goldstein LJ (2004). Prognostic and predictive factors in early-stage breast cancer. Oncologist.

[32] Beenken SW, Urist MM, Zhang Y, Desmond R, Krontiras H, Medina H, Bland KI (2003). Axillary lymph node status, but not tumor size, predicts locoregional recurrence and overall survival after mastectomy for breast cancer. Ann Surg.

[33] Lee HJ, Park IA, Song IH, Kim SB, Jung KH, Ahn JH (2015). Comparison of pathologic response evaluation systems after anthracycline with/without Taxane-based neoadjuvant chemotherapy among different subtypes of breast cancers. PLoS One.

[34] Kishan AU, McCloskey SA (2016). Postmastectomy radiation therapy after neoadjuvant chemotherapy: review and interpretation of available data. Ther Adv Med Oncol.

[35] Chapman CH, Jagsi R (2015). Postmastectomy radiotherapy after neoadjuvant chemotherapy: a review of the evidence. Oncology (Williston Park).

[36] Cain H, Macpherson IR, Beresford M, Pinder SE, Pong J, Dixon JM (2017). Neoadjuvant therapy in early breast Cancer: treatment considerations and common debates in practice. Clin Oncol (R Coll Radiol).

[37] Huang EH, Tucker SL, Strom EA, McNeese MD, Kuerer HM, Buzdar AU (2004). Postmastectomy radiation improves local-regional control and survival for selected patients with locally advanced breast cancer treated with neoadjuvant chemotherapy and mastectomy. J Clin Oncol.

[38] Buchholz TA, Tucker SL, Masullo L, Kuerer HM, Erwin J, Salas J (2002). Predictors of local-regional recurrence after neoadjuvant chemotherapy and mastectomy without radiation. J Clin Oncol.

